# Supplementary material for: Causal Relationship between Aging and Anorexia Nervosa: A White-Matter-Microstructure-Mediated Mendelian Randomization Analysis
Source: Biomedicines. 2024 Aug 16;12(8):1874. doi: 10.3390/biomedicines12081874 (PMC11351342; doi:10.3390/biomedicines12081874)
Supplement: Supplementary file 1 [file biomedicines-12-01874-s001.zip › Supplementary Figure/Figure S4/Singe SNP plot of MR analysis for TL to ubm-b-2069.pdf]

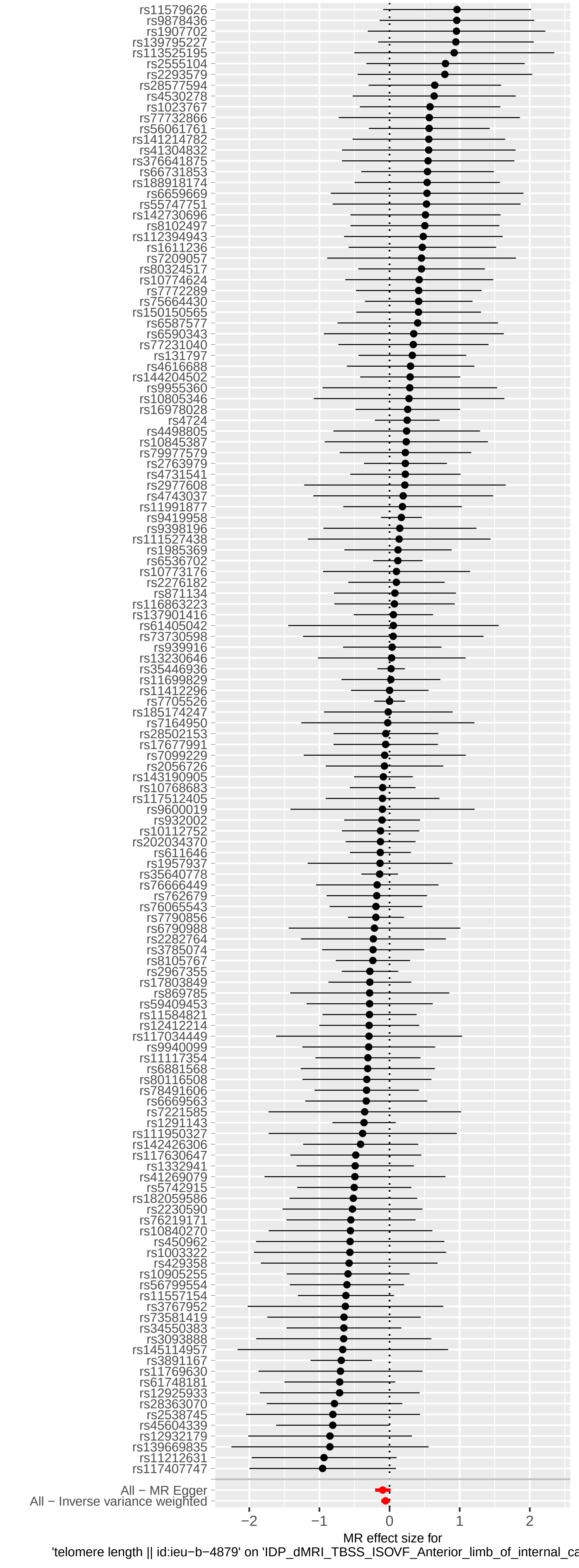

rs11579626
rs9878436
rs1907702
rs139795227
rs113525195
rs2555104
rs2293579
rs28577594
rs4530278
rs1023767
rs77732866
rs56061761
rs141214782
rs41304832
rs376641875
rs66731853
rs188918174
rs6659669
rs55747751
rs142730696
rs8102497
rs112394943
rs1611236
rs7209057
rs80324517
rs10774624
rs7772289
rs75664430
rs150150565
rs6587577
rs6590343
rs77231040
rs131797
rs4616688
rs144204502
rs9955360
rs10805346
rs16978028
rs4724
rs4498805
rs10845387
rs79977579
rs2763979
rs4731541
rs2977608
rs4743037
rs11991877
rs9419958
rs9398196
rs111527438
rs1985369
rs6536702
rs10773176
rs2276182
rs871134
rs116863223
rs137901416
rs61405042
rs73730598
rs939916
rs13230646
rs35446936
rs11699829
rs11412296
rs7705526
rs185174247
rs7164950
rs28502153
rs17677991
rs7099229
rs2056726
rs143190905
rs10768683
rs117512405
rs9600019
rs932002
rs10112752
rs202034370
rs611646
rs1957937
rs35640778
rs76666449
rs762679
rs76065543
rs7790856
rs6790988
rs2282764
rs3785074
rs8105767
rs2967355
rs17803849
rs869785
rs59409453
rs11584821
rs12412214
rs117034449
rs9940099
rs11117354
rs6881568
rs80116508
rs78491606
rs6669563
rs7221585
rs1291143
rs111950327
rs142426306
rs117630647
rs1332941
rs41269079
rs5742915
rs182059586
rs2230590
rs76219171
rs10840270
rs450962
rs1003322
rs429358
rs10905255
rs56799554
rs11557154
rs3767952
rs73581419
rs34550383
rs3093888
rs145114957
rs3891167
rs11769630
rs61748181
rs12925933
rs28363070
rs2538745
rs45604339
rs12932179
rs139669835
rs11212631
rs117407747
All − MR Egger
All − Inverse variance weighted
−2
−1
0
1
2
MR effect size for
'telomere length || id:ieu−b−4879' on 'IDP_dMRI_TBSS_ISOVF_Anterior_limb_of_internal_ca
